# Cold Chain Food and COVID-19 Transmission Risk: From the Perspective of Consumption and Trade

**DOI:** 10.3390/foods11070908

**Published:** 2022-03-22

**Authors:** Chao Zhang, Yanzhao Yang, Zhiming Feng, Chiwei Xiao, Ying Liu, Xinzhe Song, Tingting Lang

**Affiliations:** 1 Institute of Geographic Sciences and Natural Resources Research, Chinese Academy of Sciences, Beijing 100101, China; zhangc.18b@igsnrr.ac.cn (C.Z.); fengzm@igsnrr.ac.cn (Z.F.); xiaocw@igsnrr.ac.cn (C.X.); liuy.20b@igsnrr.ac.cn (Y.L.); songxz_21b@163.com (X.S.); langtt.19b@igsnrr.ac.cn (T.L.); 2 College of Resources and Environment, University of Chinese Academy of Sciences, Beijing 100049, China; 3 Faculty of Geographical Science, Beijing Normal University, Beijing 100875, China; 4 Key Laboratory of Carrying Capacity Assessment for Resource and Environment, Ministry of Natural Resources, Beijing 100101, China

**Keywords:** cold chain food, COVID-19 transmission, contaminated foods, complex network analysis, food security

## Abstract

Since the outbreak of the coronavirus disease 2019 (COVID-19), political and academic circles have focused significant attention on stopping the chain of COVID-19 transmission. In particular outbreaks related to cold chain food (CCF) have been reported, and there remains a possibility that CCF can be a carrier. Based on CCF consumption and trade matrix data, here, the “source” of COVID-19 transmission through CCF was analyzed using a complex network analysis method, informing the construction of a risk assessment model reflecting internal and external transmission dynamics. The model included the COVID-19 risk index, CCF consumption level, urbanization level, CCF trade quantity, and others. The risk level of COVID-19 transmission by CCF and the dominant risk types were analyzed at national and global scales as well as at the community level. The results were as follows. (1) The global CCF trade network is typically dominated by six core countries in six main communities, such as Indonesia, Argentina, Ukraine, Netherlands, and the USA. These locations are one of the highest sources of risk for COVID-19 transmission. (2) The risk of COVID-19 transmission by CCF in specific trade communities is higher than the global average, with the Netherlands–Germany community being at the highest level. There are eight European countries (i.e., Netherlands, Germany, Belgium, France, Spain, Britain, Italy, and Poland) and three American countries (namely the USA, Mexico, and Brazil) facing a very high level of COVID-19 transmission risk by CCF. (3) Of the countries, 62% are dominated by internal diffusion and 23% by external input risk. The countries with high comprehensive transmission risk mainly experience risks from external inputs. This study provides methods for tracing the source of virus transmission and provides a policy reference for preventing the chain of COVID-19 transmission by CCF and maintaining the security of the global food supply chain.

## 1. Introduction

The recent COVID-19 event related to frozen foods has caused public concern about the cold food chain (CCF) [1]. Many studies have focused on the impact of the pandemic on the food supply chain [2] and the external supply of food [3]. Since the outbreak of COVID-19, there have been continuous studies of the transmission path [4]. Air-borne transmission through breathing is a widely accepted route [5]. To our knowledge, the study of the seafood market in Wuhan, China initially considered seafood to be a potential carrier of viruses, and SARS-CoV-2 has been detected in imported CCF [6,7]. As a result, the questions of whether SARS-CoV-2 is foodborne and whether the food supply chain is a transmission path for the virus have aroused public concern and scholarly attention [8].

Testing for COVID-19 is a challenge because there is a lack of reliable, inexpensive, and easy-to-use detection tools, specifically for food [9]. Laboratory studies have shown that COVID-19 can remain highly stable under refrigeration at 4 °C, and in freezing conditions from −10 to −80 °C, on fish, meat, poultry, and swine skin, for 14–21 days [10,11,12]. From July to August 2020, at least 9 cases of live SARS-CoV-2 were reported being detected on the surface of frozen food in China, triggering a new round of COVID-19 spread in Beijing and Qingdao [11]. Particularly, in the Beijing Xinfadi epidemic (June, 2020), 45 people in close contact with the CCF environment were tested positive in Xinfadi Market [1]. In the Qingdao epidemic (September 2020), two positive patients are the stevedores engaged in CCF, both carried out loading and unloading of frozen cod in bulk. Gene sequencing results show that: the virus genome sequence of the stevedores is highly homologous to the original piece from the frozen cod outer package, with two distinct nucleotides. Once the destination is reached, the COVID-19 may infect stevedores or other staff working in the port through direct contact with the goods according to Liu and his colleague [13]. Lately, in the latest round of the COVID-19 in Dalian, China (in early November 2021), 16 of the 45 positive patients were workers in designated cold storage for imported CCF.

These findings indicate that frozen food is an important and potential source of the outbreak of COVID-19 [14]. In China, many batches of CCF have been detected for surviving COVID-19 (Table S1). At least eight cases of the COVID-19 spread are related to the imports of CCF in 2020. All the zero patients had contact with the contaminated cold chain food and they were all cold chain practitioners. All the SARS-CoV-2 sources can be traced from the outer packages or container samples of the cold chain (Table 1). In some countries with significant COVID-19 transmission rates, such as in the USA, Canada, Brazil, Germany, and Ireland, many production and processing workers in the CCF environment have been infected and have transmitted COVID-19 [15,16,17,18]. If there is COVID-19 infection in a certain part of a processing plant among processing personnel, cross-contamination in the CCF supply chain environment may occur, increasing the probability of COVID-19 transmission [18,19,20] (Table S2).

Meanwhile, there is also evidence of a correlation between the survival environment of COVID-19 and the cold chain transportation environment in international trade [10,21], providing environmental conditions that could allow SARS-CoV-2 to be transported through the international cold chain [17]. COVID-19 may be transported and exported on frozen food packaging over a long period of time. Cold chain transportation has the environmental attribute of low temperatures throughout the process, allowing COVID-19 to remain stable, prolonging its survival time, and increasing the difficulty of elimination [22]. There is research evidence that cold chain transportation in the frozen food industry may have caused a recurrence of COVID-19 cases in the destination (Table S2) [13,23,24]. In high-risk areas of the COVID-19 pandemic, all links of the CCF production and marketing chain could be contaminated by people with latent and asymptomatic infections [25].

Based on the existing research and our analysis, we hypothesize that meat and meat products, dairy products, fruits, vegetables, ready-to-eat food, and other cold chain transport food may be potential carriers and transmitters of COVID-19 [24,26,27]. Different parts of the CCF supply chain, such as harvesting, processing, transportation, and retail, have physical environments that may spread infections. Contaminated food in the cold chain has become a focus of COVID-19 transmission research in food chain circulation [11] and reflects the possibility that COVID-19 can be transmitted from farm to table, highlighting a source of virus transmission between countries.

The continuous increase in globalization has increased the intensity of food trade links between countries, leading the global food system to become increasingly integrated. There has been a gradual increase in the dependence of countries with weak food production capacity on external food supplies, with an increased demand for transnational long-distance food transportation [28]. Urbanization leads to the formation of large-scale urban agglomerations. As a result, local agricultural systems cannot provide enough food to meet the demand, and cities increasingly rely on food distribution systems outside the region. Cross-regional food allocation has become important in supporting the food system of big cities. 

The acceleration of globalization and urbanization has also changed food consumption demands, including the rise of fast-food culture (e.g., McDonald’s and KFC), and an increase in the demand for fresh food (e.g., fruits, vegetables, and aquatic products). Food consumption in different places and the increased demand for the convenient preservation of food had led to increased requirements for food transportation, advancing the development of cold chain transportation [29]. Cold chain transportation, with its properties of low temperature and preservation, plays an important role in local-to-local food flow and global food trade and plays an increasingly important role in daily life [22]. It has become an indispensable part of the food supply chain, particularly in urban areas. As a result, the spread of the virus through the cold chain between and within countries could heighten local food safety risks around the world across the trade network [30].

In countries with weak COVID-19 detection capability, the transmission of COVID-19 through CCF may not be found in time and may not receive adequate attention [20]. Some scholars believe that cold chain food is a potential and non-negligible transmission route in the occurrence and development of COVID-19 [31,32], but few studies on the risk of viral transmissions through the CCF trade [33]. Despite this, the scientific identification of global CCF COVID-19 transnational infection sources, and the risk of transmissions in the global CCF chain, is important for cutting off COVID-19 transmission through CCF and maintaining the security of the food supply chain [26,34]. Therefore, by applying the perspective of CCF consumption levels and the global CCF trade network (CCFTN), this study explores the risk level of COVID-19 transmission by CCF. The study combines the COVID-19 risk index (*CRI*), CCF consumption level, and trade level of different countries to address the following questions: (1) What are the trade network patterns and core node characteristics of the global CCFTN? (2) What is the “source” of the intensity pattern of COVID-19 transmission by CCF in the trade network? (3) What is the risk level of SARS-CoV-2 transmission by CCF in different countries? What are the differences in dominant risk factors? What are the regional differences?

Based on the global CCF consumption quantity and trade flow under general conditions, as well as the current COVID-19 risk of different countries, here, CCF consumption quantity and trade flow pattern and the risk of COVID-19 transmission by CCF were studied. We used consumption and trade matrix data from the Food and Agriculture Organization of the United Nations (FAO), specifically on meat, eggs, milk, vegetables, and fruits. Nutritive conversion factors (NCF) were used to convert the trade quantity into energy (kcal) equivalent. Based on an analysis of the CCF consumption level, the complex network analysis (CNA) method was used to analyze the CCF trade network features and to identify the core nodes. To identify the risk “source” of COVID-19 transmission by CCF, a COVID-19 transmission model for CCF was constructed, including the risk level, consumption level of CCF, urbanization rate (UR), and trade quantity of CCF in different countries. The risk level of CCF transmission was evaluated across the globe—trade communities—country dimensions, and the dominant risk types of different countries were identified. This study provides a scientific basis for preventing COVID-19 transmission through the cold chain, scientifically preventing and controlling the risk of transmission, and protecting the security of the global food supply chain.

## 2. Methods and Data Processing

**Figure 1:**
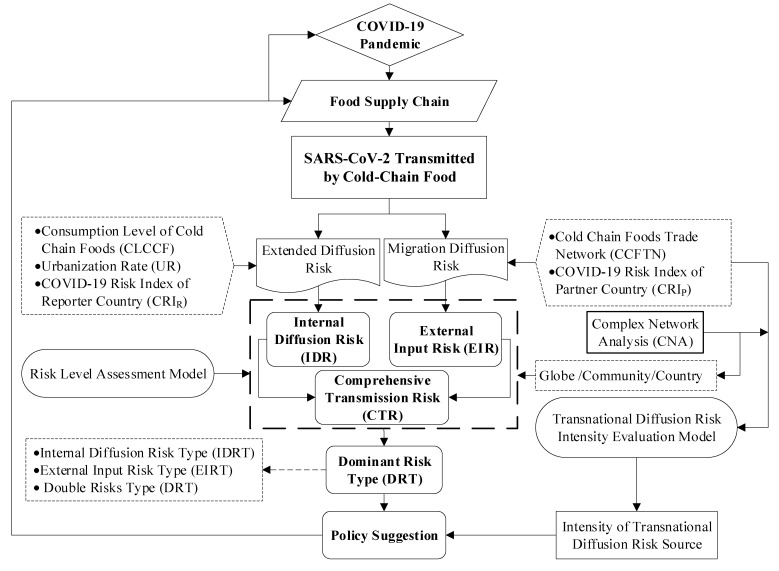
Study framework of COVID-19 transmission risk by CCF.

Considering the quantity of food consumption in cold chain and the flow of international trade network, this study builds on the following research framework (Figure 1), including analysis of CCFTN characteristics—, evaluation of COVID-19 diffusion source intensity—, Assessment of COVID-19 transmission risk level—, classification of dominant risk types, and—Policy recommendations”. The study starts with two perspectives, i.e., internal diffusion and migration diffusion. First, the internal diffusion risk (IDR) was evaluated based on the UR, CCF consumption level [35], and domestic *CRI*. Second, the CNA method was used to analyze the characteristics and community structure pattern of global CCFTN; this method has been widely applied to study trade characteristics and virus spread [36,37]. The global migration diffusion source intensity was then analyzed, based on the out-strength degree and *CRI* level. Third, the external input risk (EIR) was evaluated based on CCF imports, the number of partners, and the *CRI* of partner countries. Based on the IDR and EIR, the comprehensive risk assessment and dominant risk type identification were determined using different dimensions: globe, community, and country. Finally, proposes adaptive strategies to prevent the risk of COVID-19 transmission through CCF and maintain the security of the global food supply chain.

### 2.1. Methods

#### 2.1.1. Cold Chain Food Trade Network Analysis

(1) Network properties

Based on the CCF trade network, we analyzed the characteristics of the CCFTN using three indicators that are closely related to COVID-19 transmission: node degree, node strength, and betweenness centrality. 

Node degree ($K_{i}$) measures the number of contacts maintained by each node. Here, it represents the number of trade partners a country has. The node in-degree counts the number of links incoming to a node; it is measured by

(1) $$K_{i}^{in}=\sum_{j=1}^{N}a_{ji}$$

The node out-degree counts the number of links emanating from a node; it is measured by

(2) $$K_{i}^{out}=\sum_{j=1}^{N}a_{ij}$$

where *a* is an element of CCFTN. Generally speaking, the higher the $K_{i}^{in}$ and $S_{i}^{in}$ of a country, the higher the risk of migration diffusion risk. The higher the $K_{i}^{out}$ and $S_{i}^{out}$ of a country, the higher the intensity of transnational diffusion risk. 

Node strength ($S_{i}$) is the weighted corollary to the node degree. It measures the sum of the weights for nodal links. Node in-strength sums the value of links incoming to a node; it is measured by

(3) $$S_{i}^{in}=\sum_{j=1}^{N}w_{ji}$$

The node out-strength sums the value of links emanating from a node; it is measured by

(4) $$S_{i}^{out}=\sum_{j=1}^{N}w_{ij}$$

where *w* is an element of weighted CCFTN. If a country has a higher value of $K_{i}^{out}$ and $S_{i}^{out}$, it indicates the country has a larger number of export partners and volumes. By extension, the country has a higher level of influence in the CCFTN. In contrast, a country with a high value of $K_{i}^{in}$ and $S_{i}^{in}$ indicates that the country has a larger number of import partners and volumes [38].

The Betweenness Centrality (BC) of a node is defined as a number of shortest paths through the node. BC is measured as follows:

(5) $${\mathrm{BC}}_{i}=\frac{1}{N^{2}}\sum_{i,t}\frac{n_{si}^{i}}{g_{si}}$$

In the equation, $g_{si}$ is the number of shortest paths from node *s* to node *i*; and $n_{si}^{i}$ is the number of shortest paths through node *i* in the $g_{si}$ shortest paths from node *s* to node *i.* A higher BC value for a node indicates that the node is more important in the CCFTN, and that there is a higher level of COVID-19 transmission efficiency by CCF [37].

(2) Community detection

Several independent and highly connected modules (i.e., communities) generally form in a complex network; the connection strength between countries in the same community is higher than the connection strength with different communities. To better analyze the source of EIR, we identified the community structure of the CCFTN. The specific calculation formula of modularity is as follows:

(6) $$Q=\frac{1}{2m}\sum_{ij}(A_{ij}-\frac{k_{i}k_{j}}{2m})\partial (c_{i}c_{j})$$

In the equation, $c_{i}$ and $c_{j}$ represent the communities in which the *i* and *j* nodes are located, respectively. The variable ∂ represents the binary functions for estimating whether two points are in the same community. If $c_{i}=c_{j}$, then the value is 1; otherwise, the value is 0. The variable $A_{ij}$ is the weight of the connection between nodes *i* and *j*. The expression

(7) $$k_{i}=\sum_{j}A_{ij}$$

is the sum of all connection weights that contain node *i*. The expression

(8) $$m=\frac{1}{2}\sum_{ij}A_{ij}$$

is the total contact weight of the entire network, where the modularity is a standardized index, and the value interval is (−1, 1). In this study, the Modularity index is used to evaluate the degree of community separation. Q represents the Modularity index, and its value ranges from (0, 1). The larger the value, the more obvious the differentiation of network community and the higher the quality of community division [39].

#### 2.1.2. Analysis of the Intensity of COVID-19 Transmission Sources 

We constructed the intensity of the COVID-19 transnational diffusion index (*CTDI*) to evaluate the probability that COVID-19 occurs as an output. This is defined as follows:

(9) $$CTDI_{i}=S_{i}^{out}\times CRI_{i}\;$$

If a country has a high value of CTDI, it indicates that the country has a higher probability and range of COVID-19 transmission through the CCFTN to other countries.

#### 2.1.3. Risk Assessment Model of COVID-19 Migration Diffusion 

As noted above, COVID-19 transmission risk was analyzed from the perspective of internal diffusion and external input. The internal diffusion risk index (*IDRI*) is defined as:

(10) $$IDRI_{i}=CLCCF_{i}\times UR_{i}\times CRI_{i}$$

where $CLCCF_{i}$ represents the CCF consumption level of country *i*; $UR_{i}$ represents the *UR* of country *i*; and $CRI_{i}$ represents the COVID-19 risk index of country *i*. Generally speaking, the higher the level of urbanization, the higher the demand for CCF transfer and transportation. The greater the consumption of CCF, the higher the probability that residents will be exposed to CCF. Therefore, the higher the level of urbanization, CCF consumption, and the risk of the COVID-19, the higher the internal diffusion risk of COVID-19 transmission by CCF.

Next, the external input risk index (*EIRI*) is defined as:

(11) $$EIRI_{i}=\sum_{j}IQ_{j}\times CRI_{j}$$

where $IQ_{j}$ represents the quantity of CCF imported from country *j* to country *i*; and $CRI_{j}$ represents the COVID-19 risk index of country *j*. Correspondingly, the greater the import volume, the higher the risk of the COVID-19 risk in the country of the import pattern country, and the higher external input risk. Next, we build a comprehensive transmission risk index (*CTRI*) using *IDRI* plus *EIRI*, as follows:

(12) $$CTRI_{i}=IDRI_{i}+EIRI_{i}$$

The range in values of *IDRI* and *EIRI* strongly and equally influence *CTRI*; therefore, we applied the Jenks breaks in ArcGIS 10.5 to divide *IDRI* and *EIRI* into 10 levels, assigning the numbers 1–10 according to the level from low to high.

Finally, the contribution rate of *IDRI* and *EIRI* to *CTRI* was used to classify the dominant risk types. This was calculated based on the proportion of *IDRI* and *EIRI* scores to *CTRI*. If the contribution rate of *IDRI* was more than 50%, the country was classified as having an internal diffusion risk type (IDRT). If the contribution rate of *EIRI* was more than 50%, the country was classified as having an external input risk type (EIRT). If the contribution rate of *IDRI* and *EIRI* is equal, the country is classified as having a double risks type (DRT). The criteria for classification of all different elements (i.e., *IDRI*, *EIRI*, *CTRI*, *CFF* Consumption, and *UR*) above list in Table 2.

### 2.2. Data Processing

#### 2.2.1. Cold Chain Food Data

Our study selected 175 items in these five foods (see Table S3), namely meat, eggs, milk, vegetables, and fruits. In order to make the statistical data of consumption quantity and trade flow of different types of CCF comparable as well as better reflect the spatial differences of consumption level and trade flow of CCF, the nutritive factors were used. Through nutritive factors, the consumption and trade volume of CCF are uniformly converted into energy (kcal) to reflect its quantity [40]. Since the global cold chain food consumption-trade data have not yet been released during the COVID-19 pandemic, we use the latest data that can be found, which are the data from 2018, to present the global CCF consumption and trade situation under normal circumstances. The metadata of food trade and consumption were collected from the Trade and Food Balance database of FAOSTAT (http://www.fao.org/faostat/en/#data, accessed on 20 May 2021). In particular, missing data were replaced and supplemented by the conversion of import and export data, under the premise that one country’s export is another country’s import. For example, when querying import data, if the import data for a reporting country were missing for the partner country, the missing data were collected by querying the export data of other partner countries exporting to that reporting country (e.g., the FAOSTAT “Partner” and “Reporter” countries). After this data supplementation step (needed for about 30 countries), trade data for 198 countries were finally obtained. Network analysis and visualization were conducted using Gephi (version 0.9.2, https://gephi.org/, accessed on 1 June 2021).

#### 2.2.2. COVID-19 Risk Index Data

The COVID-19 risk index data were collected from the website at the http://covid19-risk-index.com/ (accessed on 15 May 2021); the data were compiled by teams at the Huashan Hospital of Fudan University in China and from other institutions. To mitigate the impact of data fluctuations, the average values from 1 January to 30 April 2021, were used If data were missing for the countries in the study sample, we extrapolated the data based on the average of their sub-regions (using United Nations classification standards). Finally, the data of *UR* (urban population of the total population) in 2018 were collected from the World Bank.

## 3. Results

### 3.1. Consumption Level and International Trade Network Characteristics of Cold Chain Food

#### 3.1.1. Consumption Level and Urbanization Rate

**Figure 2:**
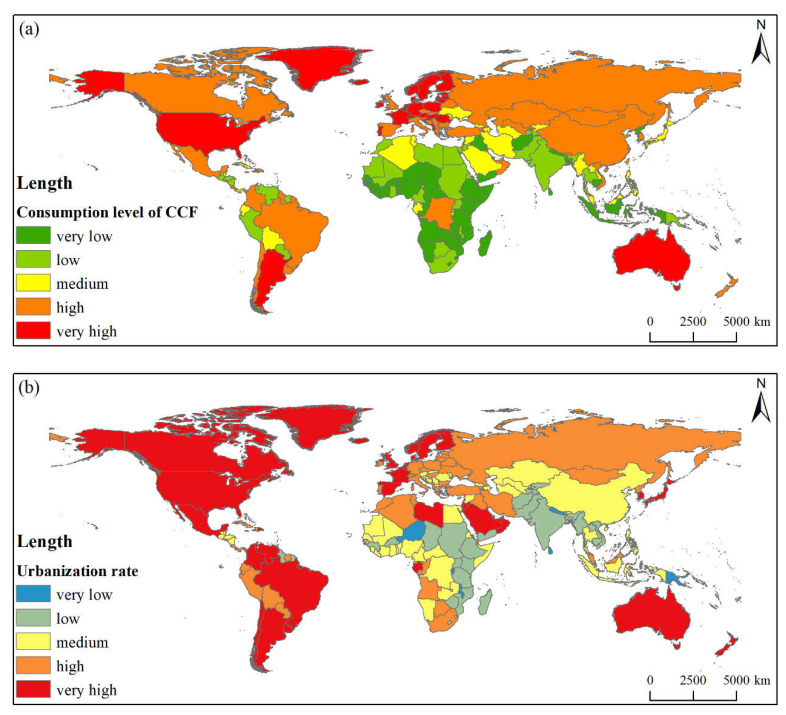
Spatial patterns of CCF consumption level (**a**) and urbanization level (**b**).

From the perspective of CCF consumption patterns, Figure 2a shows that 31 countries belong to very high consumption levels, such as European countries, the USA, Argentina, Australia and some European countries. A total of 51 countries are classified as having a high consumption level, mainly distributed in Americas and Asia. Moreover, a total of 116 countries are classified as having a medium consumption level and below, mainly in Africa, West Asia, Southeast Asia, and Southeast Asia. From the perspective of the urbanization level and distribution pattern, the countries in Europe and America are classified as very high and high; the countries in Southeast Asia, West and Central Africa, and the Southeast Asian islands are generally classified as having a medium level; and the land countries in South Asia and Southeast Asia are generally classified as having a low and very low level (Figure 2b).

#### 3.1.2. International Trade Network and Community Structure

(1) International trade network characteristics

**Figure 3:**
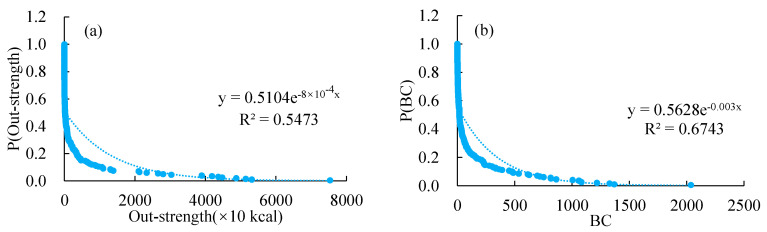
Probability distribution of strength-out degree (**a**) and BC (**b**).

The CCFTN has 198 nodes and 11,924 lines, with an annual trade quantity of 80,249.05 × 10^10^ kcal. The probability distribution fitting results illustrating different attributes of the nodes in the cold chain transportation network show that the out-strength distribution of nodes in CCFTN has typical scale-free distribution characteristics with strong heterogeneity (Figure 3a). Export core nodes play a leading role in the structure and normal operation of the trade network.

**Figure 4:**
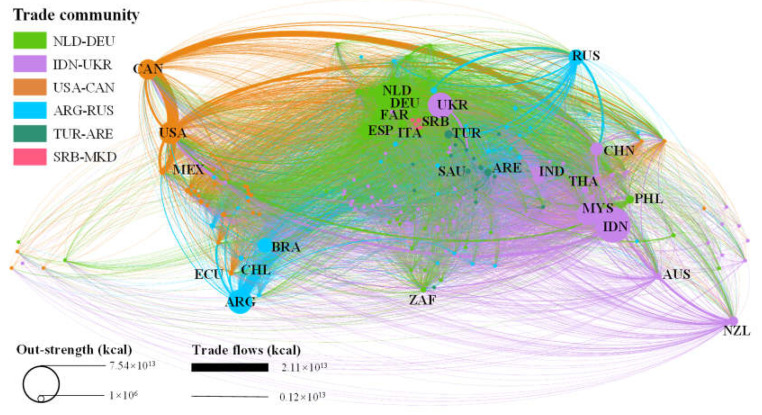
Community structure and flow patterns in the CCFTN.

In terms of trade flow (Table 3 and Figure 4), the top 20 countries in the CCFTN account for 66.06% of the export quantities; with the exception of the out-degree of Papua New Guinea, Bolivia, and Belarus, which are lower than 70, the out-degree of other top 20 countries exceed 100. According to Table 3, the top 20 countries account for 77.40% of the world’s total out-strength, with the top 10 countries accounting for 56.86% of the globe’s totality. With respect to the trade quantity and out-strength, the top 10 countries affect the CCFTN pattern, given their large export quantities and relatively extensive external links. These countries also form the core node of the trade network and the transition point of COVID-19 risk. The average BC value for the top 20 countries is 902.68 (Table 3 and Figure 3b), which is 6.59 times the global average; and the average value of the out-degree is 164, which is 2.72 times the global average. The countries with high and middle BC levels have an important position in a trade hub, and profoundly impact the global cold chain transport trade network structure. Therefore, such countries are more likely to become potential nodes for transmitting COVID-19.

(2) Community structure

The results of community structure analysis show that the CCFTN includes 6 trade communities, dominated by big exporting countries (Figure 4). The total quantity of intra-community trade is 48,477.35 × 10^10^ kcal, accounting for 60.37% of the trade across the total network (Table 4). Community division results and internal energy flow characteristics are as follows (Figures S1 and S2). (1) The quantity of trade for the NLD-DEU community accounts for 28.43% of the total CCFTN. The Netherlands, Germany, Spain, Belgium, and France are the main exporting countries; CCF mainly flows to Germany, the Netherlands, Italy, France, Belgium, and other European countries, and also radiate to parts of Africa. (2) The quantity of trade for the IND-UKR community accounts for 15.07% of the total CCFTN. CCF mainly flows from Indonesia, Ukraine, Malaysia, and New Zealand to India, China, and Malaysia, radiating to Southeast Asia, Australia, and Africa. (3) The quantity of trade for the USA-CAN community accounts for 8.54% of the total CCFTN. The USA and Canada are the main exporting countries. CCF mainly flows into each other between the USA and Mexico as well as Canada. South Korea, Japan, and other American countries are important CCF export destinations. (4) The quantity of trade for the RUS-ARG community accounts for 6.33% of the total CCFTN. Russia, Argentina, Brazil, and Belarus are the main CCF outflow sources. CCF flows mainly to Russia, Algeria, Egypt, Iran, Norway, Venezuela, and some South American, Central Asia, and West Asia countries. (5) The quantity of trade for the TUR-ARE community accounts for 1.85% of the total CCFTN. CCF mainly flows from the United Arab Emirates, Turkey, and Saudi Arabia to Iraq, Yemen, the United Arab Emirates, and other Western Asian countries. (6) The quantity of trade for the SRB-MKD community accounts for 0.15% of the total CCFTN, with generally singular flows of CCF from Serbia to the other four countries.

### 3.2. Risk Assessment of COVID-19 Transmission by Cold Chain Food

**Figure 5:**
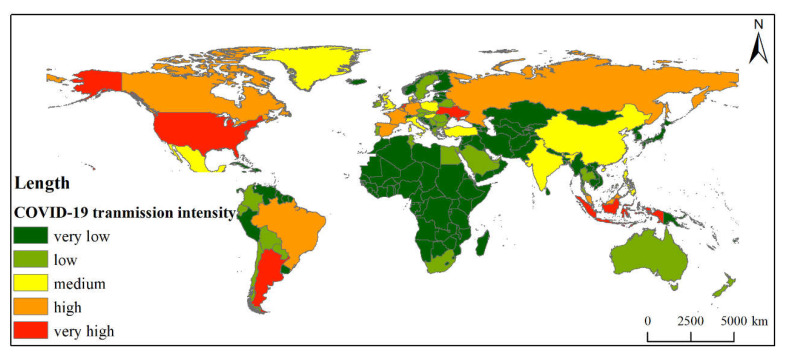
National of COVID-19 intensity transmission by CCF.

The results of transnational diffusion risk intensity evaluation showed that 5 countries are associated with very high-risk levels (Figure 5), namely, Indonesia, Argentina, Ukraine, Netherlands, and the USA. There are 8 countries classified at high risk level, and 11 countries are at a medium risk level; 27 and 147 countries are classified as having low and very low risk levels, respectively, accounting for 13.64% and 74.24% of the total (see Figure 5 and Figure S3).

### 3.3. COVID-19 Transmitted by Cold Chain Simulation

#### 3.3.1. Spatial Pattern of Internal Diffusion Risk 

The results of the IDR assessment show that the average *IDRI* is 24.63 globally, with significant differences among trade communities and countries. For specific trade communities (Table 4), the averages of the SRB-MKD and NLD-DEU IDR indices are about 1.5 and 2 times the global average, respectively; the IDR within the communities is high. The averages of the USA-CAN, TUR-ARE, and ARG-RUS community IDR values are 25.93, 25.16, and 23.32 respectively, which are close to the global average; the IDR within the communities is not high. The IDR value in the IDN-UKR community is only 11.12, which is lower than the global level.

**Figure 6:**
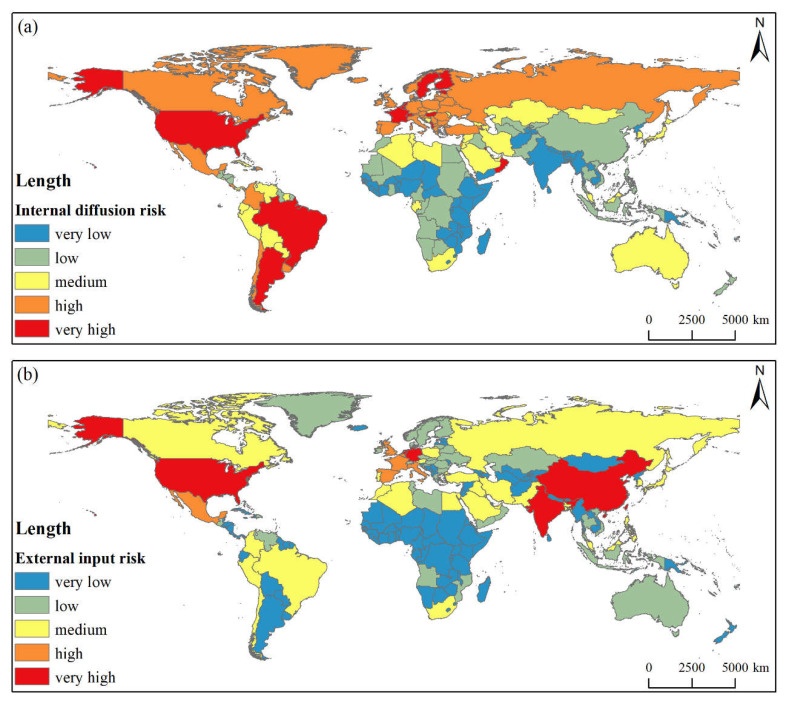
Spatial pattern of internal diffusion risk (**a**) and external input risk (**b**).

From the perspective of national scale (Figure 6a and Table 5), 19 countries are classified as having a very high IDR level, mainly in Europe, but also including the USA, Argentina, Brazil, Oman, Kuwait, and other countries. A total of 37 countries are classified as having a high IDR level, mainly in Europe. There are 40 countries with medium IDR levels and 49 with low IDR levels scattered in Asia and Africa, respectively. There are 53 countries with very low levels of IDR, mainly distributed in Africa, but also including India, Pakistan, and some Southeast Asian countries.

#### 3.3.2. Spatial Pattern of External Input Risk 

The results of the EIR assessment show that the average global *EIRI* is 23.01. The *EIRI* of the NLD-DEU and USA-CAN communities are higher than the global average level, at 36.57 and 23.81, respectively. The EIR indices of the IDN-UKR, ARG-RUS, and TUR-ARE communities range between 15–20, lower than the global average. The *EIRI* of the SRB-MKD community is only 4.63, reflecting a low level of external risk (Table 4).

There are 5 countries (i.e., Indonesia, China, the Netherlands, the USA, and Germany) with high *EIRI*; this also includes the major CCF importing countries and BC core countries. There are 7 countries with high-level *EIRI*, including Italy, Britain, France, Belgium, Spain, Mexico, and Bangladesh. Countries with medium and low EIR levels account for 12.12% and 16.67% of the total number of countries, respectively. The countries with medium risk are mainly distributed in West Asia and South America. The countries with low-level risk mainly include European countries. Approximately 65% of the countries in the world have a very low EIR level; these are mainly distributed in Africa (Figure 6b and Table 5).

#### 3.3.3. Spatial Pattern of Comprehensive Risk

The CTR assessment showed that the average value of global CTR index is 9.24. For each community, except for the IDN-UKR the average of the other five communities (i.e., the NLD-DEU, the USA-CAN, the ARG-RUS, the TUR-ARE, the SRB-MKD) is higher than the global average. Of these 5 communities, the NLD-DEU group has the highest CTR index, followed by SRB-MKD; the CTR index of the two communities was about 11.5. These are followed by TUR-ARE, USA-CAN, and ARG-RUS, with CTR indices ranging between 9.5 and 9.9 (See Table 4).

**Figure 7:**
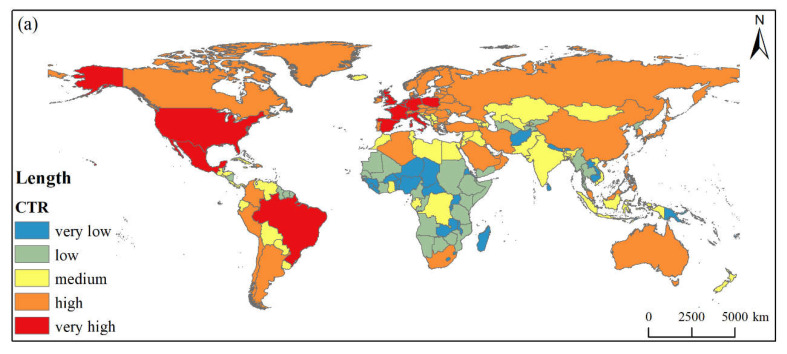

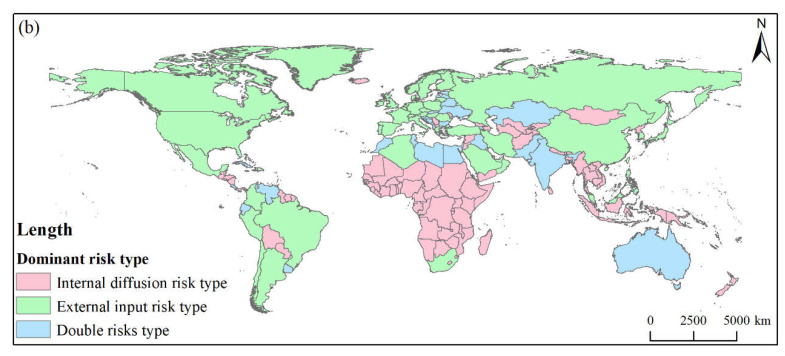
Spatial pattern of comprehensive risk (**a**) and dominant risk type (**b**).

From a country-level perspective, 11, 45, 51, 51, and 40 countries have CTR indices from very high level, to high, to middle, to low, to very low level, respectively. The few countries with a very high CTR level include the Netherlands, the USA, Germany, Belgium, France, Spain, Britain, Italy, Mexico, Poland, and Brazil. There is little difference in the indices across the other CTR levels. In terms of spatial distribution, European and American countries generally have very high and high CTR levels (Figure 7a). The CTR index of countries in Africa is low, with the exception of Algeria and South Africa. In Central Asia, South Asia, and Southeast Asia, the CTR level is medium or lower. Some countries in Western Asia, China, the Philippines, Malaysia, and Australia are at a high CTR level (See Figure 7a and Table 5).

### 3.4. Types of Dominant Risk 

The identification of dominant risk types revealed that 61.62% of countries are dominated by IDR, with 22.73% of countries are experiencing primarily external input risks. Also, some a few countries face double risks, and accounting for 15.66% of the total country count. From a spatial distribution perspective (Figure 7b), the IDR types are mainly distributed in Africa, Southeast Asia, Central Asia. In contrast, North America, South America, and most parts of Europe are dominated by external risk. A few countries in Eastern Europe, North Africa, and South Asia are dominated by double risks.

## 4. Discussion

There have been instances of COVID-19 spread related to CCF events, and there is a chance that CCF is a carrier that facilitates the transmission [14]. This study uses 2018 global matrix data of CCF consumption and trade, NCF, and a CAN method to identify the core nodes and communities of the global CCFTN. The study also explored the potential source intensity for COVID-19 transmission by CCF. The risk assessment model evaluated the extended diffusion and migration diffusion risk of COVID-19; the variables included the *CRI*, UR level, CCT consumption level, CCF import volumes, and *CRI* of exporting countries. The risks associated with COVID-19 transmission based on CCF trade were evaluated at three different dimensions i.e., national, and global scales, as well as community level. This led to the identification of the dominant risk types.

The main contributions of this study are as follows. (1) CNA method was applied to analyze global CCFTN patterns and core nodes. (2) The risk of COVID-19 through CCF was analyzed from two perspectives: local-to-local extended diffusion, and country-to-country migration diffusion. The dominant risk type was also identified for different countries. Our research could provide a scientific basis for preventing the transmission COVID-19 by CCF and maintaining the global food supply chain and food safety. Also, another contribution of this study is that it focused on the unique context of the outbreak events related to CCF. CCF has special requirements related to the storage environment, making it a unique research object. Within this unique context, factors such as UR, the consumption level of CCF, the import quantities of CCF, and the *CRI* of importing partner countries were all considered to comprehensively evaluate the risk of COVID-19 transmission in CCF. In contrast to previous studies on cereal or commodity trade, the study describes global CCF trade pattern characteristics from the perspective of physical quantities of food, using the NCF and CNA method.

Our study found that Indonesia, Argentina, Ukraine, Netherlands, the USA, and other countries have a high level of intensity with respect to CCF migration diffusion sources. The NLD-DEU community, USA-CAN community, countries importing CCF, and core countries with a high BC level (e.g., Indonesia, China, Netherlands, the USA, and Germany) face a high EIR. There is a high level of CCF consumption and urbanization in Europe and North America. The SRB-MKD and NLD-DEU communities, European countries, the USA, Argentina, Brazil, Oman, and Kuwait, face high IDR.

In terms of the CTR, the CTR level of most trade communities is higher than the global average, with the NLD-DEU community having the highest levels. The CTR levels of 11 countries are at a very high level, including 8 European countries (namely, Netherlands, Germany, Belgium, France, Spain, Britain, Italy, and Poland) and 3 American countries (i.e., the USA, Mexico, and Brazil). The European region faces a high CTR level, with most countries dominated by ETI. Globally, IDR dominates the transmission risk of COVID-19 by CCF in many countries (accounting for about 62% of all countries, mainly in African countries), followed by the countries with high levels of EIR (accounting for about 23% of the countries); fewer countries faced both risks (internal and external) (approximately 15% of the countries).

There are three key results highlighted here. Firstly, the CCFTN has typical scale-free network characteristics, and the stability of the trade network is significantly affected by the large trading countries. Given this, stable CCF output from large exporting countries significantly impacts the CCFTN. The COVID-19 pandemic negatively affected global food production, processing, and transportation. Stable CCF exports also significantly impact the maintenance of the global CCF supply chain; while large CCF exports also increase the probability of COVID-19 transmission [7]. Therefore, the large CCF exporting countries have become the key nodes for maintaining global food trade security and transnational export controls during the COVID-19 pandemic. At the same time, due to the functions served by transfer hubs, the countries with high and middle BC levels may become transfer hubs for COVID-19 during food importation. This occurs through the import and export of contaminated processed food. This is risk associated with countries having a BC value more than 1000, which includes the USA, the Netherlands, Britain, Malaysia, Canada, France, Belgium, and China. These countries are key nodes of food transfer in the global cold chain and could be core hubs of COVID-19 transnational spread—and for cutting the viral transmittal off. 

Secondly, after years of development, CCF has become an important part of the global food supply chain, and the security of CCF impacts global food security. Once the pandemic began, it was inevitable that COVID-19 would impact the global food supply chain [7]. The world now faces increased risks from farm and food supply chain interruption, with increased risks of unemployment and food insecurity in food-related fields. The interruption of the food supply chain also increases global food waste. Cold chain technology reduces food loss and improves the efficiency of the food supply chain and food quality standards [35]. During the COVID-19 pandemic, managing CCF inventories has become an important way to address food shortages [41,42,43]. 

However, cold chain technology also increases the scope of food trade circulation, improves food accessibility and availability. During the COVID-19 pandemic, quarantine protocols have increased people’s demand for CCF in their homes [44]. Compared with non-cold food chain transportation, CCF faces the dual pressure of controlling COVID-19 transmission and maintaining a smooth food supply chain [11]. Therefore, strengthening monitoring of the virus and maintaining the normal circulation of CCF is of great significance for maintaining food security. This deserves increased attention from policymakers [45].

Thirdly, this study found that internal diffusion risk is the dominant risk for many countries with respect to possible COVID-19 spread by CCF. It is vital to strengthen virus monitoring and daily protection of CCF workers to prevent and control COVID-19. The virus can be transmitted through respiration, contact, and other routes; as such, food contamination may occur during CCF production, processing, storage, transportation, and retail. As such, virus infection may occur at any point in the CCF life cycle, from farm to fork [11]. This highlights the possibility that the transmission chain may extend from human infection to food pollution back to human infection. 

In terms of environmental factors (cold and humidity) and operational design (workers standing side-by-side on crowded and noisy processing lines), the situation in meat processing plants has been shown to facilitate the spread of the virus (Table S2). The crowded living conditions of temporary foreign workers in fruit and vegetable production operations can also support virus transmission and spread [46,47]. The risk associated with CCF is higher compared to non-CCF because of these unique operational variables. The COVID-19 pandemic has increased the risk of global unemployment, and cold chain workers face higher risks of infection while also facing unemployment risks [48]. They may also not report medical concerns, exacerbating the risk of virus transmission [49]. This also deserves more attention and again highlights the need to strengthen monitoring tools for detecting COVID-19 transmission in the cold chain environment, and to introduce daily prevention and control practices for workers involved in the cold chain [4]. These steps would help maintain the normal operation of the cold chain food system and prevent and control future transmissions.

Countries with a strong source intensity value with respect to COVID-19 migration diffusion should work to strengthen their load-level monitoring of exported CCF, to prevent transmission through contaminated food. Countries with a high *EIRI* that import CCF should also strengthen their monitoring of imports to prevent COVID-19 transmission. We acknowledge that this testing recommendation is challenging, due to the lack of a reliable, inexpensive, and easy-to-use COVID-19 detection tool specifically for food [9]. We recommend, therefore, that international organizations develop guidelines for COVID-19 detection in CCF and apply inter-disciplinary methods to develop new tools of prevention [5]. It is also important to assist countries that have an insufficient detection capacity to gradually eliminate COVID-19 transmission. 

There are five countries (i.e., Indonesia, Argentina, Ukraine, Netherlands, and the USA) with a very high level of diffusion risk intensity. Those five countries are the countries of global concern in preventing COVID-19 transmission by CCF trade networks. The country with high import quantity and in-degree and high BC (e.g., India, China, Netherlands, the USA, Germany) facing high-level external input risk. If lack of detection ability of COVID-19, those countries can easily become a transmission hub for global COVID-19 diffusion by CCF trade network. So, those countries are the countries of special concern in COVID-19 diffusion in the world. 

Most countries with the high consumption level of CCF are faced with high comprehensive transmission risk, and most of them belong to external input risk types (mainly European countries). On the one hand, these countries need to strengthen the COVID-19 prevention and control in the processing, transportation, and preparation of CCF, popularize the knowledge of prevention in CCF, and reduce the risk of infection through contact with CCF. On the other hand, it is necessary to strengthen the port transshipment of CCF and the storage management of imported CCF, cut off the chain of external input risk in time, and maintain the security of CCF and the food supply chain.

For the countries that belong to the internal diffusion risk type (mainly in Africa), their CCF COVID-19 detection ability is insufficient. We suggest that international organizations should give priority to strengthening assistance to enhance the COVID-19 detection intensity of their CCF and reduce the risk of internal diffusion.

Also, some relevant studies showed that the lack of reliable, cheap, and easy-to-use COVID-19 detection tools for food was still one of the challenges [9,50,51], and we then suggest that global medical institutions and enterprises strengthen the research and development of cold chain food virus monitoring. We also believed that policymakers formulate technical guidelines and disinfection for SARS-CoV-2 prevention in processing-packaging-loading and uploading(stevedore)-retailing-cooking of CCF, to guide residents to scientifically handle imported CCF in daily life and reduce the risk of COVID-19 epidemic caused by exposure to CCF.

It is worth nothing that our study had two main methodological limitations. (1) The data were limited to the data in the FAO Database. As such, we included only 5 kinds of food that need cold chain transportation. These foods do account for most CCF and are generally representative of that food category. However, we were not able to obtain good data about aquatic product trade quantities. As such, future studies could focus on aquatic products as the research object. Future research could mine aquatic data and physical conversion parameters to study the risk associated with SARS-CoV-2 transmission in aquatic products.

It should be noted that our study did not take the trade data after the COVID-19 outbreak into consideration due to accessibility. It is pointed out that after the outbreak, the international trade will be affected in a short time, and the trade volume will decrease, while the domestic CCF will increase with the implementation of the isolation policy [2,46,52,53], which may affect the accuracy of the results of this study. This study is based on the general CCF consumption and trade volume. Considering that the COVID-19 pandemic is continuing, a comparative study of CCF and the risk of SARS-CoV-2 transmission needs to be carried out around the COVID-19 Outbreak in the near future.

(2) Different countries are in different development stages of cold chain transportation, and the prevention and control measures for COVID-19 differ across the CCF flow process. Although the comprehensive COVID-19 risk index (e.g., *CRI*) covers the factor of prevention and control measures, the research did not more granularly detect COVID-19 in the CCF process flow. This may affect the accuracy of the assessment results. Additional industry data about cold chain industry development could improve the accuracy of the risk index and improve the risk assessment of COVID-19 transmission by CCF. In addition, future studies would be helpful in validating that the incidences of COVID-19 transmission have occurred across the hypothesized communities, to validate or refute the model in real-life contexts. The lack of actual testing to validate the model is an acknowledged research gap that deserves further exploration [9,50,51]. As we all know, the difference of food matrix will also affect the survival time of virus [26]. Future research can focus on the spread of CCF viruses from the perspective of food matrix.

## Figures and Tables

**Table 1:** Some case of imported cold food supply chain-related coronavirus disease cold chain practitioners.

Outbreak Time	Outbreak City	Place Cases (Tested)	Zero Patient	SARS-CoV-2 Source
11 June 2020	Beijing	Agricultural products in 335 (>11,000,000) Xinfadi Wholesale market	Employee	Environmental swab samples related to imported salmon
22 July 2020	Dalian, Liaoning	Kaiyang company (Seafood 92 (>6,000,000) processing enterprises) Dock	Dockworker	Outer packaging of imported fish
24 September 2020	Qingdao, Shandong	Qingdao port, Dock 2 (5781)	Stevedores, Dockworker	Outer packaging surface of imported frozen cod
11 October 2020	Qingdao, Shandong	Darang company of Qing-12 (10,920,000) dao port	Stevedore Dockworker	Outer packaging of imported frozen cod
25 October 2020	Kashgar, Xinjiang	Kashgar Airport 138 (4,746,500)	Stevedore	Container from abroad
7 November 2020	Tianjin	Hailian Frozen Food Co., 12 (1,030,000) Ltd Tianjin Hailian cold storage	Stevedore Dockworker	Outer packaging of imported pig food packaging of frozen pork
9 November 2020	Shanghai	Pudong Airport 4 (>14,000)	Stevedore	Airborne container from North America
15 December 2020	Dalian, Liaoning	Dalian Port Yidu cold chain 83 (6,379,000) Co., Ltd.	Stevedore	Environmental swab samples related to imported cold chain food

Sources: Health Times (www.jksb.com.cn, accessed on 10 September 2021); Chinese Center for Disease Control and Prevention (www.chinacdc.cn, accessed on 10 September 2021).

**Table 2:** Criteria for classification of different elements.

Level	Jenks Breaks				Equal Interval
	*IDRI*	*EIRI*	*CTRI*	CCF Consumption	*UR*
Very Low	0–8.15	0–1.05	1–4	0–386	0–20
Low	8.15–19.67	1.05–3.19	5–8	386–605	20–40
Medium	19.67–35.78	3.19–8.14	9–12	605–834	40–60
High	35.78–56.18	8.14–15.99	13–16	834–1154	60–80
Very high	56.18–91.34	15.99–42.81	17–20	1154–1870	80–100

Note: The original values of *IDRI* and *EIRI* are very large, so the *IDRI* and *EIRI* are divided by 10^5^ and 14^10^.

**Table 3:** Characteristics of the top 20 countries in the CCFTN.

Export Quantity				Out-Strength			Betweenness Centrality		
ISO3	Value (10^10^ kcal)	Proportion (%)	Outdegree	ISO3	Value (10^10^ kcal)	Proportion (%)	ISO3	Value	Outdegree
IDN	43.57	7.34	173	IDN	7537.79	9.39	USA	2037.71	187
ARG	36.38	6.13	134	NLD	5316.55	6.63	NLD	1368.40	186
UKR	34.28	5.78	150	UKR	5142.20	6.41	GBR	1327.77	161
NLD	28.58	4.82	186	ARG	4875.42	6.08	MYS	1217.67	184
RUS	26.92	4.54	104	USA	4476.27	5.58	CAN	1082.32	152
CAN	25.62	4.32	152	MYS	4367.64	5.44	FRA	1072.42	179
USA	23.94	4.03	187	DEU	4184.12	5.21	BEL	1059.53	183
MYS	23.74	4.00	184	CAN	3894.32	4.85	CHN	1005.16	187
DEU	23.64	3.98	177	ESP	3034.50	3.78	DEU	863.48	177
ESP	17.24	2.91	176	RUS	2799.30	3.49	ESP	815.76	176
BRA	14.66	2.47	181	BRA	2653.96	3.31	SAU	813.87	123
BEL	12.76	2.15	183	BEL	2334.45	2.91	NZL	752.39	161
PNG	12.23	2.06	15	CHN	2127.72	2.65	AUS	719.76	133
FRA	11.75	1.98	179	FRA	2104.12	2.62	ITA	697.17	177
CHN	11.38	1.92	187	NZL	1384.96	1.73	ZAF	626.26	156
BOL	10.24	1.73	35	ITA	1315.44	1.64	ARE	615.10	141
BLR	9.13	1.54	65	POL	1304.79	1.63	IND	534.72	171
PHL	8.90	1.50	120	TUR	1100.14	1.37	UKR	488.39	150
NZL	8.60	1.45	161	DNK	1087.44	1.36	CHE	478.08	119
POL	8.36	1.41	156	PHL	1068.04	1.33	TUR	477.70	170
Top20	391.94	66.06	/	Top20	62,109.18	77.40	/	/	/
Global	593.33	100.00	/	Global	80,249.05	100.00	/	/	/

Note: ISO3 is the nation code, see the Table S4 for the full country name. “/” indicates no corresponding data.

**Table 4:** Characteristics of the CCFTN community structure.

Name	Intra-Community Trade		Node		Edge	*CRI*	*IDRI*	*EIRI*	*CTRI*
	Quantity (10^10^ kcal)	Proportion (%)	Number	Proportion (%)	Number				
1-NLD-DEU	22,817.73	28.43	51	25.76	1395	7.29	38.16	36.57	11.88
2-IDN-UKR	12,089.99	15.07	60	30.30	972	3.23	11.12	19.04	6.23
3-USA-CAN	6854.80	8.54	30	15.15	371	6.13	25.93	23.81	9.67
4-ARG-RUS	5080.57	6.33	35	17.68	394	5.31	23.32	15.65	9.57
5-TUR-ARE	1485.12	1.85	17	8.59	137	5.53	25.16	15.43	9.88
6-SRB- MKD	119.15	0.15	5	2.53	20	8.60	48.20	4.63	11.40
Global	80,249.05	/	198	100.00	11924	5.42	24.63	23.01	9.24

Note: The original values of *IDRI* and *EIRI* are very large; as such, the *IDRI* and *EIRI* are divided by 10^5^ and 14^10^.

**Table 5:** Classification statistics of different COVID-19 transmission risks.

Level	Internal Diffusion Risk		External Input Risk		Comprehensive Risk	
	Number	Proportion (%)	Number	Proportion (%)	Number	Proportion (%)
Very low	53	26.77	129	65.15	40	5.56
Low	49	24.75	33	16.67	51	22.73
Medium	40	20.20	24	12.12	51	25.76
High	37	18.69	7	3.54	45	25.76
Very high	19	9.60	5	2.53	11	20.20

## Data Availability

The data presented in this study are available on request to authors.

## Supplementary Materials

The following are available online at https://www.mdpi.com/article/10.3390/foods11070908/s1, Figure S1: Flow of the emery within the global CCFTN communities. A total of 198 countries are ranked according to the total trade quantity and plotted clockwise in descending order. The size of the out bar indicates the total trade quantity (unit: 106 kcal). Export quantity is indicated with links emanating from the out bar of the same color. (a) NLD-DEU community, (b) IDN-UKR community, (c) USA-CAN community, (d) ARG-RUS community, (e) TUR-ARE community, (f) SRB-MKD community, Figure S2: Spatial pattern of trade community of cold chain foods trade networks (CCFTN), Figure S3: Spatial pattern of the COVID-19 risk index (*CRI*), Table S1: Imported cold-chain food tested positive for SARS-CoV-2 in China in 2020, Table S2. Cold-chain food tested positive for SARS-CoV-2 in countries in 2020, Table S3: The cold chain food products and the calorie factors, Table S4: The country name and ISO3 code.
