# Supplementary material for: Cold Chain Food and COVID-19 Transmission Risk: From the Perspective of Consumption and Trade
Source: Foods. 2022 Mar 22;11(7):908. doi: 10.3390/foods11070908 (PMC8998142; doi:10.3390/foods11070908)
Supplement: Supplementary file 1 [file foods-11-00908-s001.zip › Supporting Information_Revision.pdf]

Supporting Information for

Cold Chain Food and Covid-19 Transmission Risk: From the Perspective of Consumption and Trade

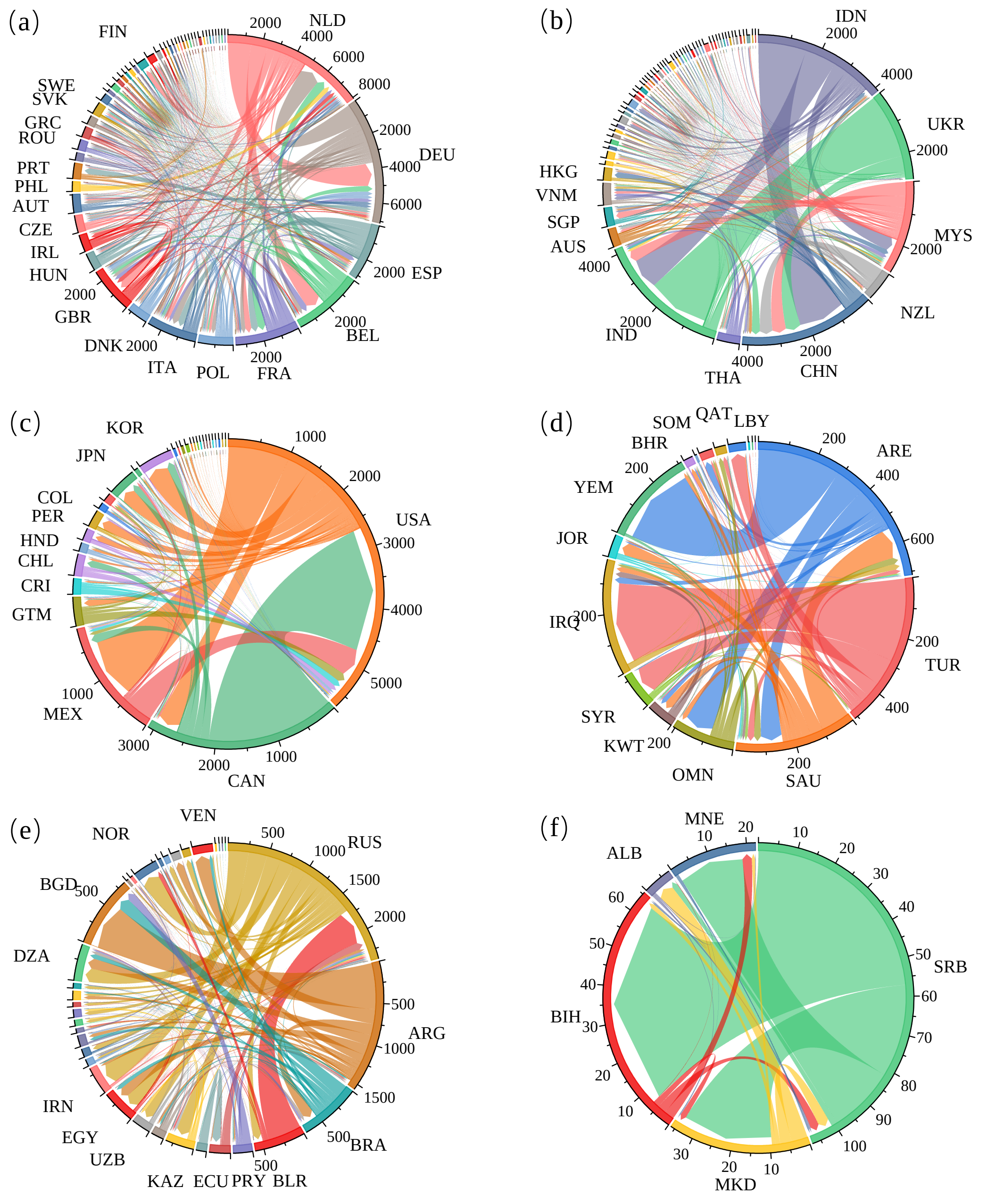

Figure S1 Flow of the emery within the global CCFTN communities. A total of 198 countries are ranked according to the total trade quantity and plotted clockwise in descending order. The size of the out bar indicates the total trade quantity (unit: 10^6 kcal). Export quantity is indicated with links emanating from the out bar of the same color. (a) NLD-DEU community, (b) IDN-UKR community, (c) USA-CAN community, (d) ARG-RUS community, (e) TUR-ARE community, (f) SRB-MKD community.

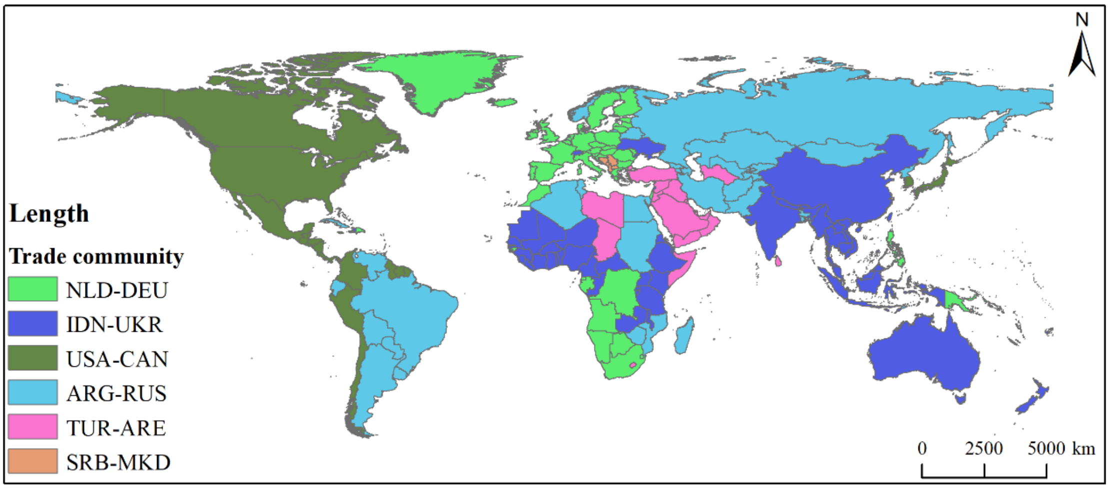

Figure S2 Spatial pattern of trade community of cold chain foods trade networks.

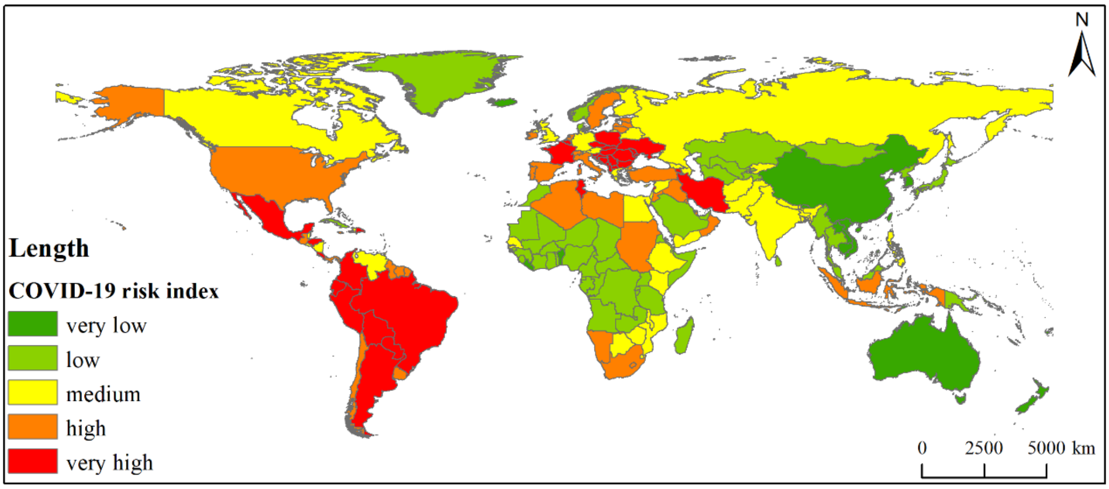

Figure S3 Spatial pattern of the COVID-19 risk index (CRI)

Table S1 Imported cold-chain food tested positive for SARS-CoV-2 in China in 2020 [1]

Date	Product	From	Found by	Note
June 11	Frozen salmon	Not reported	Xinfadi market (XFDM), Beijing city	SARS-CoV-2 was found on frozen seafood in China for the first time.
July 3 to August 13	Frozen white shrimp	Ecuador	Dalian, Xiamen, and five other cities in Shandong Province	For the second time, SARS-CoV-2 was found on frozen seafood in China following a month after the COVID-19 outbreak in Beijing.
August 9	A variety of frozen seafood	Not reported	Dalian in Liaoning Province, Yantai in Shandong Province	
August 13	Frozen chicken wings	Brazil	Guangdong Province	
September 18	Frozen hairtail	Indonesia		

September 23	Frozen deep-water redfish	Norway		
September 24	Frozen cod outer package's surface	Not reported	Qingdao city, Shandong Province	This is the first time worldwide, SARS-CoV-2 was isolated from the frozen fish outer package's surface. The virus has not been isolated due to the low nucleic acid concentration of the samples (from the outer package's surface) tested.
September 25	Frozen hairtail	Brazil		
October 1	Frozen boneless beef	Brazil		
October 17	Frozen cod	Not reported	Qingdao Port, Shandong Province	
October 31	Frozen pomfret	Ecuador		
November 6	Aquatic product	Russia and Netherlands		
November 7–8	Frozen pork and frozen hairtail	Germany and India	Tianjin city	No community transmission has occurred.
November 10	Frozen pomfret	Indonesia		
December 9	Frozen boneless beef	Brazil		
December 17	Frozen cod	Not reported	Dalian in Liaoning Province	No community transmission has occurred.

Table S2 Cold-chain food tested positive for SARS-CoV-2 in countries in 2020

Time	Outbreak city	Place	Number infected/Ratio%	Reference
As for May 31, 2020	United States of America, 14 States	Meat and poultry processing	16233/9.1%	[2]
June 12, 2020	United States of America, Oregon	Seafood plant	132/ Not reported	[3]
June 17, 2020	Germany, Gütersloh	Slaughterhouse	657/66.66%	[4]
June 08, 2020	Germany, Westfleisch	Slaughterhouse	151/75.5%	[5]
June 23, 2020	United Kingdom, Anglesey	Chicken processing site	150/ Not reported	[6]
August, 2020	New Zealand, Auckland	Worker engaged in handling frozen food	4/ Not reported	[7]

References

1. Lu, L.C.; Quintela, I.; Lin, C.H.; Lin, T.C.; Lin, C.H.; Wu, V.C.H.; Lin, C.S. A review of epidemic investigation on cold - chain food - mediated SARS - CoV - 2 transmission and food safety consideration during COVID - 19 pandemic. *Journal of Food Safety* **2021**, *n/a*, e12932, doi:10.1111/jfs.12932.

2. Waltenburg, M.A.; Victoroff, T.; Rose, C.E.; Butterfield, M.; Jervis, R.H.; Fedak, K.M.; Gabel, J.A.; Feldpausch, A.; Dunne, E.M.; Austin, C.; et al. Update: COVID-19 Among Workers in Meat and Poultry Processing Facilities - United States, April-May 2020. *Mmwr-Morbidity and Mortality Weekly Report* **2020**, *69*, 887-892, doi:DOI 10.15585/mmwr.mm6927e2.

3. Pacific reopens some plants after COVID-19 outbreak; Icicle reports new cases in Alaska. Available online: https://www.seafoodsource.com/news/supply-trade/pacific-reopens-some-plants-after-covid-19-outbreak-icicle-reports-new-cases-in-alaska (accessed on

4. Coronavirus: Over 600 people test positive at German slaughterhouse. Available online: https://www.dw.com/en/coronavirus-over-600-people-test-positive-at-german-slaughterhouse/a-53846038 (accessed on

5. Coronavirus outbreak closes German meat-packing plant. Available online: https://www.dw.com/en/coronavirus-outbreak-closes-german-meat-packing-plant/a-53374478 (accessed on

6. Coronavirus: Why have there been so many outbreaks in meat processing plants? Available online: https://www.bbc.com/news/53137613 (accessed on

7. Hu, L.; Gao, J.; Yao, L.; Zeng, L.; Liu, Q.; Zhou, Q.; Zhang, H.; Lu, D.; Fu, J.; Liu, Q.S.; et al. Evidence of Foodborne Transmission of the Coronavirus (COVID-19) through the Animal Products Food Supply Chain. *Environ Sci Technol* **2021**, *55*, 2713-2716, doi:10.1021/acs.est.0c06822.
